# Generative artificial intelligence–assisted medical self-care and associated factors among undergraduate students at Arsi University, South-Eastern Ethiopia

**DOI:** 10.1371/journal.pdig.0001748

**Published:** 2026-09-24

**Authors:** Jibril Bashir Adem, Anas Ali Alhur

**Affiliations:** 1 Department of Public Health, College of Health Science, Arsi University, Asella, Ethiopia; 2 Department of Health Information Management and Technology, College of Public Health, Imam Abdulrahman Bin Faisal University, Dammam, Saudi Arabia; University College London, UNITED KINGDOM OF GREAT BRITAIN AND NORTHERN IRELAND

## Abstract

The rapid expansion of social media and the availability of Generative Artificial Intelligence (GenAI) technologies like ChatGPT and Gemini have significantly increased access to personalized health information. This has led to a rise in self-directed health behaviors such as self-diagnosis and self-medication. While GenAI can support informed self-care decisions, over-reliance on these technologies may result in incorrect self-medication and delayed consultations with medical professionals if not managed appropriately. However, there is a notable lack of systematic empirical data regarding GenAI-assisted medical self-care practices, particularly in Ethiopia. This study aimed to assess the prevalence of GenAI-assisted medical self-care and its influencing factors among undergraduate students at Arsi University in South-Eastern Ethiopia. A cross-sectional study was conducted with 414 randomly selected students using a self-administered questionnaire. Data were collected through Kobo Toolbox, and descriptive analyses alongside binary logistic regression were performed to identify factors associated with GenAI-assisted self-care. The study achieved a response rate of 97.87%, with participants having a mean age of 22.2 years. Results showed that 70.05% of students engaged in GenAI-assisted medical self-care. Factors positively associated with this behavior included being a second-year student (AOR=2.98), possessing good digital health literacy (AOR=1.71), awareness of GenAI technologies (AOR=2.18), a positive attitude towards these technologies (AOR=1.63), and recent health facility visits (AOR=2.46). Conversely, good health-seeking behavior (AOR=0.61) and infrequent health facility visits (AOR=0.49) were linked to lower odds of engaging in GenAI-assisted self-care. The findings suggest a high prevalence of GenAI-assisted self-care among university students and highlight the need for guidelines and training on digital health literacy for effective and safe technology use in healthcare.

## Author summary

Patient engagement with self-directed health behaviors, such as self-diagnosis, disease-related information seeking, and the use of self-medication as a primary strategy or as a substitute for formal medical care, has significantly increased in parallel with advancements in digital technology and Generative Artificial Intelligence (GenAI) tools. Empirical evidence suggests that health science professionals, students, and those with higher levels of education and professional accomplishments engage in higher rates of medical self-care, due to their greater access to medical information through peer networks, scientific literature, and pharmaceutical companies. We employed a cross-sectional survey among undergraduate students at Arsi University using self-administered questionnaires to assess the use of GenAI–assistance for medical self-care and its associated factors. The study demonstrated that GenAI-assisted medical self-care is highly prevalent among higher education students. The study also found that individual, technological, and behavioral factors influence the adoption of GenAI-assisted medical self-care. Given a growing dependence on innovative technologies such as large language models and GenAI, universities, healthcare professionals, and policymakers should enhance digital health literacy and provide guidelines on the appropriate and safe use of GenAI in health care, while encouraging students to seek medical advice for conditions that require clinical evaluations.

## Introduction

Utilization of alternative medical self-care and engaging in self-medication are choices made by individuals that fall within the umbrella of health care-seeking behavior [1,2]. This includes the attitudes and actions of individuals aimed at illness prevention (Primary prevention), early diagnosis and treatment of current medical conditions (Secondary prevention), and the prevention of complications related to established diseases (Tertiary prevention) [3,4]. Self-medication is the use of drugs to treat self-diagnosed symptoms or illnesses without professional medical supervision and represents a widespread phenomenon with potentially significant implications for the global health system [5]. Conceptually, it’s regarded as a subset of self-care, which constitutes a complex process including diagnostic decision-making of disease and its causes, choosing the right medicine, and ensuring the effectiveness of treatment [6]. However, in practice, self-medication is the independent use of a medicinal product, usually over-the-counter (OTC) drugs, which are that are obtained without a prescription from care providers [7,8].

Evidence indicates that the majority of illness episodes are treated by self-medication in many developing countries because of poor quality and accessibility of professional health services and skepticism about the perceived benefit of receiving professional medical care [9,10]. The critical limitation of self-medication lies in the absence of clinical evaluation by a trained medical professional, which could result in missed diagnosis, inappropriate drug use, and delays in initiating effective and timely treatments [11,12]. Moreover, improper self-medication leads to resource wastage, enhances pathogen resistance, and ultimately results in significant health risks, including adverse drug reactions, prolonged pain, and drug dependence [13,14].

Medical self-care practices range across population groups and are influenced by numerous factors, including age, gender, education, familial context, societal norms, legal frameworks, drug availability, exposure to marketing, and nature of the disease [8,9,15]. Empirical evidence indicates a higher rate of medical self-care among medical doctors, health science students, individuals with higher levels of education, and professional accomplishments [19–21]. These groups typically have more access to medical information through peer networks, scientific literature, and pharmaceutical companies, which makes it easier for them to self-diagnose and self-medicate, frequently through unauthorized access to prescriptions from health care providers. Furthermore, the “white coat effect” guarantees effortless access to medications available in pharmacies [10,13]. The practice of medical self-care in these population groups is further reinforced by increased self-confidence in one’s own knowledge of illnesses, symptoms, and medicinal treatments, as well as contextual factors like time constraints, illness-related stigma, reluctance to disturb or disappoint colleagues, fear of appearing weak or lacking medical knowledge, and worries about confidentiality [16–18].

Since the introduction of ChatGPT-3.5 in November 2022, Generative Artificial Intelligence (GenAI) has received widespread attention and has begun to have a transformative impact on the healthcare delivery system [19,20]. Concurrently, the growth of social media platforms and the larger internet ecosystem, combined with the increasing availability of GenAI tools such as ChatGPT, Gemini, and Copilot, has significantly improved the accessibility of health-related information in everyday life [21,22]. This increased access has been accompanied by a substantial rise in the engagement of patients with self-directed health behaviors such as self-diagnosis, disease-related information seeking, and the use of self-medication as a primary strategy or as a replacement for formal medical care [23–25].

GenAI systems process and synthesize large, diverse datasets, which enable them to provide tailored recommendations about symptoms, medications, preventive measures, and lifestyle modifications that foster a sense of personalized treatment among the users [26,27]. The growing innovations of GenAI transformed digital technologies into the primary sources of medical guidance and support for many people, especially healthcare professionals and students [19,28]. These technologies enhance convenience and perceived autonomy by facilitating rapid, on-demand, and often free access to health information, particularly for individuals with structural, personal, financial, or geographic obstacles to formal healthcare services [20,29].

While tailored recommendations provided by GenAI might help to provide an informed decision towards self-care, over-reliance on such information may also increase wrong self-medication, misinformation, or delay timely consultation with qualified medical professionals if not properly monitored or managed [30,31]. The growing reliance on GenAI for health-related decision-making causes pressing concerns about the accuracy, safety, and suitability of AI-generated guidance for effective self-care [29,30]. These emerging concerns highlight the need for an extensive investigation into the practice of GenAI-assisted medical self-care, particularly in middle- and low-income countries [32,33].

However, there is a significant lack of systematic empirical evidence from Ethiopia or other sub-Saharan African countries assessing the magnitude of GenAI-assisted medical self-care and associated factors. Thus, this study aims to address this critical evidence gap. The findings of the study will assist policymakers, healthcare providers, students, and technology experts in developing evidence-based policies and guiding responsible deployment and use of GenAI in health care contexts, and ensuring that emerging digital tools support safe, timely, and appropriate use of healthcare services.

## Methods

### Ethical consideration

Ethical clearance was obtained from the College of Health Sciences, Arsi University Ethical Review Committee (Reference number: CoHS/R/239/2025/2026). Study participants were informed of the study’s purpose and provided informed verbal consent. Participation in the study was voluntary. Participants who were unwilling to participate or wished to withdraw at any stage of the interview were allowed to do so without consequence. The confidentiality and privacy of the respondents were maintained.

### Study design and period

A cross-sectional survey was employed among Arsi University students using a self-administered questionnaire to assess utilization of GenAI–assisted self-care and its associated factors between January 20 and February 15, 2026.

### Study setting

The study was conducted among Arsi University students. The university was founded in October 2014 and is located in the Southeastern parts of Ethiopia [34]. The institution has approximately 10,000 students enrolled in more than 48 undergraduate and 32 postgraduate programs offered by its five colleges and one school. In 2021, the Ethiopian Ministry of Education classified the University as an Applied Science University, highlighting its commitment to applied sciences and practical training [35].

### Study population

The study’s source population included all undergraduate students enrolled in the university’s five colleges (agricultural and environmental sciences, health sciences, education and behavioral sciences, business and economics, and social sciences and humanities) and one school (law), while the study population consisted of students who fulfilled the inclusion criteria and provided consent.

### Inclusion and exclusion criteria

The study included all regular undergraduate students over the age of 18, first-year and above, who provided informed consent to participate. Students who were seriously ill during the data collection period, distance program students, and weekend students were excluded.

### Sample size and sampling procedure

To determine the sample size of the study, a single population proportion formula: n= $\frac{{{\text{Z}}_{\alpha /\text{2}}}^{\text{2}}\;(Pq)}{d^{\text{2}}}$ was used with a 95% confidence level (Zα/2 = 1.96), an estimated magnitude of GenAI-assisted medical self-care of 50% (P = 0.5, q = 0.5) (since there were no similar studies done in a similar setting), and an error margin of 5% (d = 0.05); the initial sample size is found to be 384. Adding a non-response rate of 10%, the final sample size of the study was determined to be 423.

A stratified random sampling method was used to choose the sample departments from the five colleges and one school in the institution. Twelve departments (medicine, pharmacy, public health, animal science, veterinary science, agricultural economics, curriculum studies and teachers’ professional development, educational planning and management, accounting and finance, marketing management, economics, and sociology and social work) were randomly selected. Simple random sampling was then used to choose study participants from each department based on the proportional sample allocation.

### Variables of the study

The outcome variable of the study is utilization of GenAI–assisted medical self-care, which is dichotomized as (yes, no). Medical Self-care practice was considered as the diagnosis, medications, and treatment without prior medical consultation with health care providers on indications, dosage, and duration of treatment [36].

The predictor variables included sociodemographic and economic factors such as age, gender, year of study, field of study, monthly income/family monthly income, religion, domicile, and region; personal and behavioral factors including khat chewing, alcohol drinking, internet/social media addiction, and smoking. Technology-related factors include computer literacy and perceived level of computer use, phone/internet use, and level of information search on social media, GenAI use and purpose, as well as clinical and health service-related factors such as disease history, health-seeking behavior, history of health facility visit, level of health services satisfaction, distance to health facility, and perceived ease of access.

### Data-collection tools and procedures

A self-administered, structured questionnaire developed based on the reviews of related studies on GenAI-assisted medical self-care practice among students was used to collect data [37–42]. The data were collected by two trained BSC nurses and supervised by one MPH public health professional, with close supervision and facilitation by the principal investigator. Data were collected digitally using Kobo Toolbox (https://ee.kobotoolbox.org/x/DkeC3a9q) on smartphones or tablets to ensure efficiency, reduce errors, and facilitate real-time data monitoring.

### Data quality assurance

The questionnaire was originally developed in English and subsequently translated into Amharic and Afaan Oromo for students with difficulty understanding the English language (S1 Text 1). A pretest was conducted with 5% of the calculated sample size from students in unsampled departments to assess clarity, language appropriateness, and content relevance. The principal investigator and supervisor provided on-site supervision during this process. Before the actual data collection, the supervisors oriented the students on the overall procedure for completing the questionnaire. The collected data were promptly reviewed by the investigators for completeness and clarity immediately after submission by the students.

### Data analysis

Data were exported to STATA version 17 for cleaning and further analysis. Descriptive analyses, such as percentages, ratios, frequency distributions, measures of central tendency, and dispersion, were conducted and presented using tables, graphs, and diagrams. A binary logistic regression was fitted using bivariate analysis (at p-value ≤ 0.25 and 95% confidence interval) to identify candidate variables and multivariable analysis (at p-value ≤ 0.05) to assess and adjust for confounding and to identify factors associated with the use of GenAI-assisted medical self-care. The Hosmer-Lemeshow test was used to evaluate the model’s goodness-of-fit.

## Results

### Sociodemographic characteristics of the study participants

The study included 414 undergraduate students, with a response rate of 97.87%. The mean age of the participants was 22.23 (SD ± 2.0), with the majority (66.43%) being above the age of 21 years. Most of the participants (70.05%) were males, and a majority (96.1%) were single and third-year students (35.3%). More than half (57.49%) of the participants were health science students with a grade point average of 3 to 3.5 (43.5%) (Table 1).

**Table 1:** Sociodemographic and economic characteristics of study participants.

Variable	Category	Frequency (n)	Percentage (%)
Age (years)	18–20	139	33.57
	>20	275	66.43
Gender	Male	290	70.05
	Female	124	29.95
Marital status	Single	398	96.14
	Married	16	3.86
Year of study	First year	44	10.63
	Second year	109	26.33
	Third year	146	35.27
	Fourth year and above	115	27.78
Field of study	Health sciences	238	57.49
	Non-health sciences	176	42.51
Grade Point Average (GPA)	<3.00	35	8.45
	3.00–3.49	180	43.48
	≥3.50	199	48.07
Monthly income (ETB)	<1,000	174	42.03
	1,000–3,000	180	43.48
	≥3,000	60	14.49
Source of income	Family support	393	94.93
	Part-time work	21	5.07
Place of upbringing	Urban	237	57.25
	Rural	177	42.75
Current living arrangement	Dormitory	401	96.86
	Off-campus	13	3.14

**Note:** Percentages are calculated based on the total sample size (N = 414). ETB = Ethiopian Birr; GPA = Grade Point Average.

### Health status, illness experience, medication history

Regarding their health state, the majority (50.24%) claimed fair health, with no known chronic diseases (94.4%). Most (62.8%) of the participants had no disease episode in the past 12 months, with about 41.6% using medication in the same period, while only 12.80% had an adverse drug reaction (Table 2).

**Table 2:** Health status, illness experience, and medication history of the study participants.

Variable	Category	Frequency	Percentage
Self-reported health status	Poor	57	13.77
	Fair	208	50.24
	Good	149	35.99
Known chronic health condition	No	391	94.44
	Yes	23	5.56
Number of illness episodes in the past 12 months	None	260	62.80
	One - two	132	31.88
	Three or more	22	5.31
Medication use in the past 12 months?	No	242	58.45
	Yes	172	41.55
Adverse drug reaction	No	361	87.20
	Yes	53	12.80

### GenAI-assisted medical self-care

**Fig 1:**
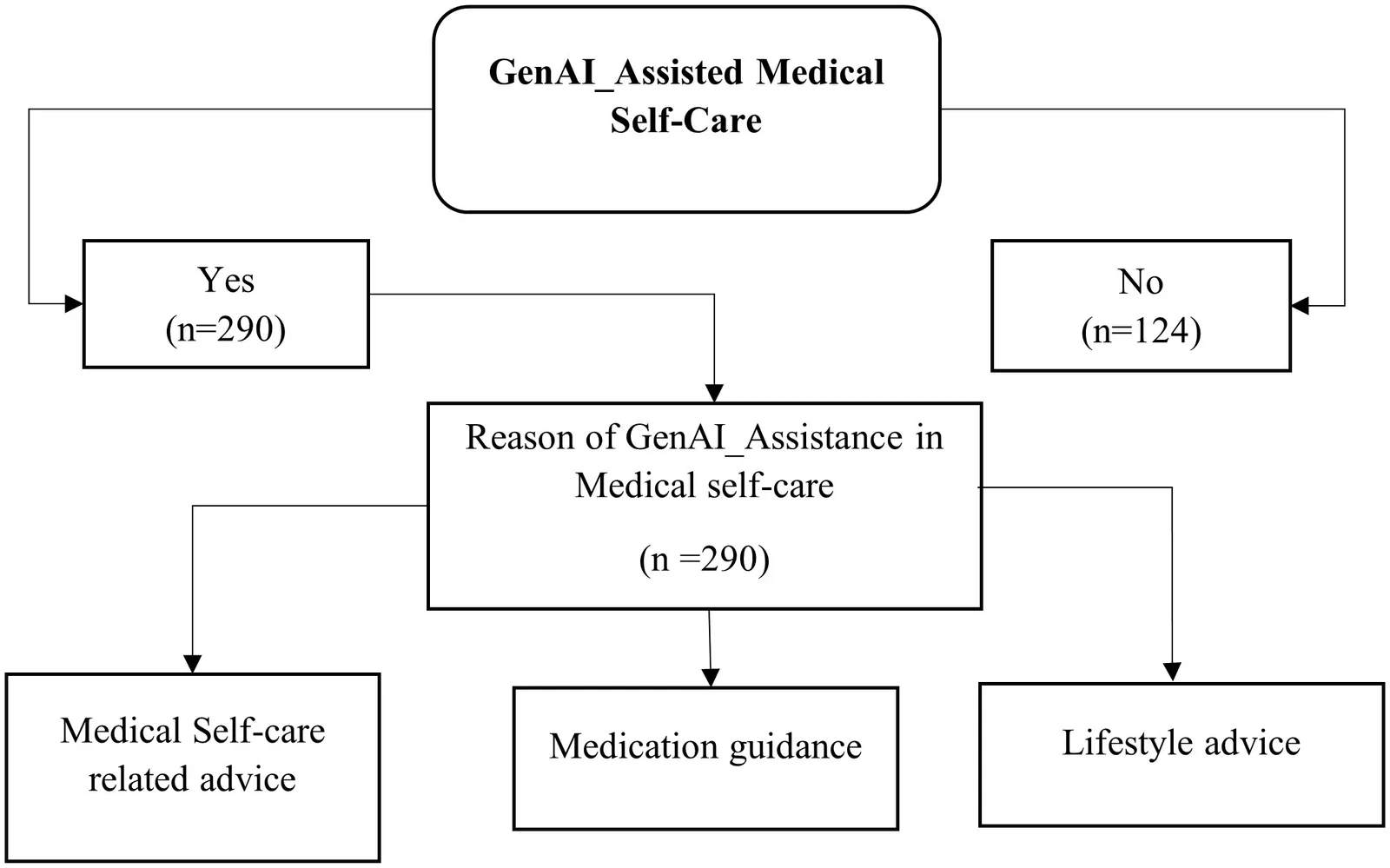
GenAI-assisted medical self-care and reason for the GenAI assistance among undergraduate students at Arsi University (n = 414).

In this study, 70.05% (95% CI: 65.64% – 74.46%) of students reported practicing GenAI-assisted medical self-care. Among the users, 73.4% used generative AI mostly for general medical self-care advice; some of them also sought medication-related information (14.5%) and lifestyle advice (12.1%) (Fig 1).

### Distribution of GenAI-assisted medical self-care utilization across participant characteristics

To assess potential stratification patterns and identify whether GenAI-assisted medical self-care utilization varied across demographic, academic, and socioeconomic groups, the distribution of GenAI use was examined using Pearson’s chi-square test. The distribution of GenAI-assisted medical self-care utilization was generally consistent across most participant characteristics. No statistically significant differences were observed by gender (p = 0.229), marital status (p = 0.219), age group (p = 0.409), field of study (p = 0.173), GPA (p = 0.320), monthly income (p = 0.470), source of income (p = 0.728), place of upbringing (p = 0.280), or current living arrangement (p = 0.948). However, GenAI-assisted medical self-care utilization differed significantly across year of study (Pearson χ² = 14.18, p = 0.003). The highest proportion of utilization was observed among second-year students (82.6%), followed by third-year students (69.9%), fourth-year and above students (63.5%), and first-year students (56.8%) (Table 3).

**Table 3:** Distribution of GenAI-assisted medical self-care utilization across sociodemographic, academic, and socioeconomic characteristics among study participants (N = 414).

Variable	Category	No GenAI-assisted medical self-care n (%)	GenAI-assisted medical self-care n (%)	Pearson χ^2^	p-value
Gender	Female	32 (25.8)	92 (74.2)	1.45	0.229
	Male	92 (31.7)	198 (68.3)		
Marital status	Married	7 (43.8)	9 (56.2)	1.51	0.219
	Single	117 (29.4)	281 (70.6)		
Age group (years)	18–20	38 (27.3)	101 (72.7)	0.68	0.409
	>20	86 (31.3)	189 (68.7)		
Year of study	First year	19 (43.2)	25 (56.8)	14.18	**0.003**
	Second year	19 (17.4)	90 (82.6)		
	Third year	44 (30.1)	102 (69.9)		
	Fourth year and above	42 (36.5)	73 (63.5)		
Field of study	Health sciences	65 (27.3)	173 (72.7)	1.86	0.173
	Non-health sciences	59 (33.5)	117 (66.5)		
Grade Point Average (GPA)	<3.00	12 (34.3)	23 (65.7)	2.28	0.320
	3.00–3.49	47 (26.1)	133 (73.9)		
	≥3.50	65 (32.7)	134 (67.3)		
Monthly income (ETB)	<1,000	50 (28.7)	124 (71.3)	1.51	0.470
	1,000–3,000	52 (28.9)	128 (71.1)		
	≥3,000	22 (36.7)	38 (63.3)		
Source of income	Family support	117 (29.8)	276 (70.2)	0.12	0.728
	Part-time work	7 (33.3)	14 (66.7)		
Place of upbringing	Rural	58 (32.8)	119 (67.2)	1.17	0.280
	Urban	66 (27.8)	171 (72.2)		
Current living arrangement	Dormitory	120 (29.9)	281 (70.1)	0.004	0.948
	Off-campus	4 (30.8)	9 (69.2)		

Note: Pearson’s chi-square test was used to assess differences in GenAI-assisted medical self-care utilization across categorical characteristics.

### Factors associated with GenAI–assisted medical self-care

Based on the observed stratification patterns and their theoretical relevance, a binary logistic regression analysis was performed to identify independent predictors of GenAI-assisted medical self-care utilization, incorporating multiple independent variables through both bivariate and multivariable analysis. In bivariate analysis, twenty predictors were examined for association with the outcome variable including marital status, gender, illness episode in the last 12 months, field of study, year of study, attitude towards GenAI technology, trust in health professionals care, availability of medicine at health facility during the last visit, affordability of last care, distance to nearest health facility, action taken whenever feel unwell, time to seek care whenever feel unwell, health facility visit for last illness, GenAI awareness, digital health literacy, internet search practice for health information, internet access, average nightly sleep, tobacco use and history of medication use.

In the multivariate logistic regression analysis, twelve variables from the bivariate analysis were considered as candidate variables at a p-value of 0.25 (this cut point was taken into consideration to avoid the early exclusion of potentially important variables and confounders) including gender, marital status, study year, field of study, illness episodes, medication usage, digital health literacy, awareness of GenAI technology, recent health facility visits, average nightly sleep, health-seeking behavior, health facility visits in the preceding 12 months, and attitudes toward GenAI technology. Seven of these variables emerged as independent predictors of GenAI-assisted medical self-care practices among participants at p<=0.05.

The likelihood ratio test showed that the final model (the model including the candidate independent variables) was significantly different from the null model (intercept-only model without explanatory variables) (LR χ² (33) = 118.42, p < 0.001), indicating that the independent variables improved the model generated from data compared to an intercept-only regression. The Hosmer-Lemeshow goodness-of-fit test indicated no poor fit (χ² (8) = 2.56, p = 0.9589), and the predicted probability closely corresponded with observed outcomes.

Accordingly, second-year students were nearly three times more likely to engage in GenAI-assisted medical self-care practice compared to first-year students {AOR of 2.98, (95% CI: 1.29–6.86, p = 0.010)}. Additionally, students with good health-seeking behavior were 39% less likely to practice GenAI-assisted medical self-care compared to those who had poor health-seeking behavior {AOR of 0.61, (95% CI: 0.38–0.98, p = 0.041)}.

Similarly, students with good digital health literacy were more likely to use GenAI-assisted medical self-care than those with poor digital health literacy {AOR = 1.71, (95% CI: 1.03-2.85, p = 0.038)}. Furthermore, students with awareness of GenAI technologies were twice as likely to engage in GenAI-assisted medical self-care as those who were not aware {AOR = 2.18, (95% CI: 1.22-3.90, p = 0.009)}, and students with a positive attitude toward GenAI technologies were 1.63 times more likely than those with a negative attitude {AOR = 1.63, (95% CI: 1.01-2.64, p = 0.031)}.

Students who attended a health facility for their recent illness were 2.46 times more likely to utilize GenAI-assisted medical self-care compared to those who did not {AOR = 2.46, (95% CI: 1.52–3.98, p < 0.001)}. Additionally, students with rare overall health facility visits during the past 12 months had a 51% lower likelihood of using GenAI-assisted medical self-care than those with regular visits {AOR = 0.49, (95% CI: 0.29-0.83, p = 0.008)} (Table 4).

**Table 4:** Bivariate and multivariable logistic regression of factors associated with GenAI-assisted medical self-care (N = 414).

Variable	Category	GenAI-assisted medical self-care		COR (95% CI)	AOR (95% CI)	P-value
		No (n = 124)	Yes (n = 290)			
Gender	Female	32 (25.8)	92 (74.2)	1.00	1.00	1.00
	Male	92 (31.7)	198 (68.3)	0.75 (0.47–1.20)	0.82 (0.49–1.36)	0.432
Marital status	Single	117 (29.4)	281 (70.6)	1.87 (0.68–5.13)	1.41 (0.46–4.29)	0.546
	Married	7 (43.8)	9 (56.2)	1.00	1.00	1.00
Study year	First	19 (43.2)	25 (56.8)	1.00	1.00	1.00
	Second	19 (17.4)	90 (82.6)	3.60 (1.66–7.81)	2.98 (1.29–6.86)	**0.010**
	Third	44 (30.1)	102 (69.9)	1.76 (0.88–3.52)	1.44 (0.67–3.07)	0.347
	Fourth+	42 (36.5)	73 (63.5)	1.32 (0.65–2.68)	1.11 (0.51–2.43)	0.786
Field of Study	Health	65 (27.3)	173 (72.7)	1.00	1.00	1.00
	Non-Health	59 (33.5)	117 (66.5)	0.75 (0.49–1.14)	0.89 (0.56–1.42)	0.633
Illness episode	None	74 (28.5)	186 (71.5)	1.00	1.00	1.00
	One–Two	41 (31.1)	91 (68.9)	0.88 (0.56–1.39)	0.92 (0.57–1.48)	0.732
	Three+	9 (40.9)	13 (59.1)	0.57 (0.24–1.40)	0.63 (0.25–1.57)	0.324
Medication use	No	79 (32.6)	163 (67.4)	1.00	1.00	1.00
	Yes	45 (26.2)	127 (73.8)	1.37 (0.89–2.11)	1.28 (0.82–2.00)	0.281
Digital health literacy	Poor	93 (34.1)	180 (65.9)	1.00	1.00	1.00
	Good	31 (22.0)	110 (78.0)	1.83 (1.15–2.94)	1.71 (1.03–2.85)	**0.038**
GenAI Technology Awareness	No	34 (47.2)	38 (52.8)	1.00	1.00	1.00
	Yes	90 (26.3)	252 (73.7)	2.51 (1.49–4.22)	2.18 (1.22–3.90)	**0.009**
Health facility visit for the recent Illness	No	79 (42.5)	107 (57.5)	1.00	1.00	1.00
	Yes	45 (19.7)	183 (80.3)	3.00 (1.94–4.65)	2.46 (1.52–3.98)	**0.001**
Average nightly Sleep	<5 hrs.	11 (30.6)	25 (69.4)	1.08 (0.51–2.28)	0.94 (0.41–2.14)	0.883
	5–7 hrs.	100 (32.2)	211 (67.8)	1.00	1.00	1.00
	>7 hrs.	13 (19.4)	54 (80.6)	1.97 (1.03–3.77)	1.62 (0.79–3.32)	0.188
Health-seeking behavior	Poor	44 (23.7)	142 (76.3)	1.00	1.00	1.00
	intermediate	70 (36.1)	124 (63.9)	0.55 (0.35–0.86)	0.81 (0.38–0.98)	0.32
	Good	10 (29.4)	24 (70.6)	0.74 (0.33–1.67)	0.61 (0.38–0.98)	**0.041**
Health facility visits in the past 12 months	Regular	41 (22.8)	139 (77.2)	1.00	1.00	1.00
	Rare	67 (44.1)	85 (55.9)	0.37 (0.23–0.60)	0.49 (0.29–0.83)	**0.008**
	No visit	16 (19.5)	66 (80.5)	1.22 (0.64–2.33)	1.11 (0.56–2.21)	0.768
Attitude toward GenAI technology	Negative	87 (33.5)	173 (66.5)	1.00	1.00	1.00
	Positive	37 (24.0)	117 (76.0)	1.59 (1.01–2.50)	1.63 (1.01–2.64)	**0.036**

Note: COR; Crude odds ratio, CI; Confidence Interval, AOR: Adjusted Odds Ratio

## Discussion

This study aims to assess the use of Generative Artificial Intelligence (GenAI)–assisted medical self-care, which is defined as using GenAI tools for self-diagnosis, medication selection, and treatment decisions without prior consultation with healthcare professionals regarding indications, dosage, and duration of treatment, and to determine the factors associated with this practice among students in higher education institutions. This study found that approximately 70.05% (95% CI: 65.64% – 74.46%) of participants engaged in GenAI-assisted medical self-care. While there is limited research conducted on the use of GenAI in terms of medical self-care globally, the result of our study is significantly higher than that in a previous study evaluating the use of GenAI-based tools for emotional or mental health needs, in which 10% used GenAI for emotional support [43].

The prevalence found in the current study was also considerably higher compared to previous study that examined traditional methods of self-care practices without considering the impact of generative AI assistance, which found 43.24% in Tabuk City [44], Saudi Arabia, 50.9% of students at King Saud University [45], and 49.3% of students at the University of Dammam [46] used self-medication In a similar vein, 12.7% of people in Spain [11], 44.5% in New Delhi [47], 47.8% in China [48], 62.9% in Egypt [49], 12.1% in Rwanda [50], 52.4% among health sciences students of University of Gondar [51], and 64.98% among undergraduate students of Wollo university, Northern Ethiopia [52]. Our finding aligns with those from previous research, showing 66% in Pakistan [53], 70% in Ghana [54], 71.7% in Nagpur, India [55], 72% among medical sciences students of Shiraz, Iran [56,57], and 73.9% in Khartoum, Sudan [57]. However, this result is lower than those reported in other studies, which indicated 77.2% in the United Arab Emirates [58], 80.45% in South India [1], 92% in West Bengal [59], and 91.4% in Southern Nigeria [60].

The higher prevalence found in the current study might be attributed to several contextual and technological factors. The first contextual attribute is that the rapid expansion and accessibility of generative AI platforms have dramatically shaped how people engage with health-related information and decision-making support [61]. Generative AI provides real-time, conversational, and personalized responses, which may encourage people (especially university students who are proficient in digital use) to use generative AI for their health-related decisions [62,63]. Secondly, digital literacy is generally high among university students, which contributes to their higher likelihood of adopting emerging digital health technologies, like AI chatbots and large language model (LLM) systems, to search for health-related information [64,65].

Thirdly, the ease, anonymity, and promptness that these tools provide when discussing minor health issues or more sensitive health-related issues are some of the reasons why university students might turn more to generative AI systems for health advice rather than traditional health care consulting methods [66]. Additionally, disparities in healthcare access, cultural perspectives on self-medication, the regulatory framework surrounding the sale of pharmaceuticals, and discrepancies in the populations examined or the techniques employed to gauge self-medical care could all contribute to the discrepancy. The rapid rise of GenAI offers a transformative shift in medical self-care; while it empowers patients with instant, personalized health information, this paradigm heavily relies on digital literacy, AI awareness, and the underlying healthcare infrastructure to ensure safe and effective adoption [67,68]. However, relying on GenAI for health decisions poses major public health risks such as misdiagnosis, delayed treatment, and misinformation; to mitigate these, young generations must develop robust digital health literacy to critically evaluate AI-generated outputs and treat these tools strictly as supplementary rather than diagnostic [69,70].

In this study, students in their second year of study were approximately three times more likely to engage in GenAI-assisted medical self-care practices compared to students in their first year. These findings are parallel to findings from a similar study at universities in Taibah (Saudi Arabia) [71], India [55,72], Pakistan [73], and Mekelle University (Ethiopia) [74], which have reported greater engagement in self-care behaviors among students beyond the first year of study. However, our findings suggest that the relationship between academic year and GenAI-assisted medical self-care is more complex than a simple progressive increase with academic advancement. Although second-year students demonstrated significantly higher utilization compared with first-year students, the magnitude of association declined among third-year and fourth-year-or-above students (AOR = 1.44 and AOR = 1.11, respectively), with confidence intervals including the null value. This pattern indicates that GenAI-assisted medical self-care adoption may increase during the early years of university education but may subsequently stabilize rather than continue increasing with academic progression.

A similar non-linear pattern has been reported among undergraduate medical students in India, where junior students demonstrated greater GenAI utilization and more favorable attitudes toward AI compared with senior students, while increasing academic seniority was associated with reduced trust in AI technologies [75]. Several explanations may account for this pattern. Students in earlier academic years may experience greater uncertainty regarding health information and may therefore rely more on easily accessible digital tools, including GenAI, to supplement their emerging knowledge and self-management skills. In contrast, students in later years may develop greater confidence in evaluating health information, have increased exposure to clinical training and professional resources, or become more aware of the limitations of large language models, including inaccurate or fabricated responses, leading to more cautious engagement with AI-based tools [75–79]. However, these explanations should be interpreted cautiously because the observed differences among third-year and senior students were not statistically significant. The findings suggest that GenAI-assisted medical self-care adoption may vary according to educational stage and learning context rather than increasing uniformly with academic progression. Future longitudinal studies should investigate how students’ knowledge, clinical exposure, trust, and perceived usefulness of AI technologies change throughout university training and how these factors influence sustained GenAI-assisted self-care practices.

Furthermore, students with poor digital literacy were less likely to practice GenAI-assisted medical self-care. This finding is consistent with previous studies indicating that digital literacy is a substantial predictor of digital health device adoption. Studies indicate that university students and online users are more inclined to seek health information on the Internet and utilize digital tools for managing their health [80,81]. Similar findings were found for college students in the United States; thus, students with higher eHealth literacy are more likely to engage in online health information seeking activities as well as digital health-related behaviors [82]. Similarly, a study conducted among university students in China found that university students with higher levels of eHealth literacy are more likely to use internet-based health resources and to assist with health-related decisions [83]. This study found that people with strong digital literacy skills can access various types of digital platforms, ask the right health-related questions, critically analyze the information displayed on these platforms, and correctly understand the responses they receive digitally. These people are more likely to employ new technology (including generative AI tools) when seeking a medical diagnosis or requesting help formulating a treatment plan. Individuals with low digital literacy skills, on the other hand, may have a more difficult time accessing and understanding health-related information digitally, making it unlikely that they will be interested in using generative AI technology to assist with their medical self-care.

Similarly, students with good health-seeking behavior were less likely than those with poor health-seeking behavior to participate in GenAI-enabled medical self-care practices. This finding is in line with other studies conducted among medical students in Bangladesh and Pakistan, which found a significant association between health-seeking behavior and self-medication [84,85]. This result supports the idea that people with poor health-seeking behavior are more likely to look for other ways to manage their health and rely on self-care techniques like self-diagnosis and self-medication rather than seeking advice from licensed medical professionals. In these studies, many students opted to manage their own illnesses without the input from a qualified health care professional.

Additionally, students with awareness and a good attitude towards Generative Artificial Intelligence (GenAI) technologies were about twice as likely to engage in GenAI-assisted medical self-care. This finding is consistent with results from previous studies conducted on the adoption of digital technology for health care delivery, which found a significant relationship between awareness, attitude toward AI, and acceptance of using AI technologies to support health service decision-making [86,87]. Furthermore, a study conducted on AI-supported health communication systems in Nigeria found that the use of AI technologies for health data, consultation, and remote healthcare delivery was significantly associated with increased awareness and familiarity with these technologies [88]. These results indicate that individuals with good awareness of GenAI technologies are more likely to see their potential advantages, gain confidence in their abilities, and then use them for health-related decision-making, such as medication selection, self-diagnosis, and treatment recommendations.

Students who attended a health facility for their most recent illness were more likely to utilize GenAI‑assisted medical self‑care, and this pattern is supported by evidence indicating that healthcare utilization influences subsequent health information–seeking and self‑management behaviors. Evidence supports the claim that healthcare utilization affects health information-seeking behavior and self-management behaviors. Studies conducted in China using nationally representative data found that higher engagement in outpatient and other formal healthcare services led to a higher likelihood of online health information seeking and using internet resources to help make decisions about their health [89]. Studies conducted in Ethiopia and elsewhere found that previous use of formal health services was significantly associated with online health information seeking and self-care activities [90,91]. These results suggest that visiting healthcare facilities may expose students to more health-related topics and lead to increased use of additional health information sources, such as digital platforms and innovative technologies like GenAI tools. Additionally, systemic factors like perceived care quality constraints, long waiting times, and limited access to advanced diagnostics and specialized care may encourage people who have had contact with formal healthcare to seek complementary or alternative assistance through digital means, increasing the likelihood that they will adopt GenAI-assisted self-care.

Recent advances in large language models have substantially improved the performance of generative AI systems in medical question answering, clinical reasoning, and decision support, with newer models demonstrating greater factual accuracy and reasoning capabilities than earlier generations [92]. Nevertheless, no current GenAI system can guarantee completely error-free clinical recommendations, and inaccurate or fabricated outputs may still occur, particularly in complex or ambiguous clinical scenarios [93]. Emerging approaches, including retrieval-augmented generation, knowledge graph integration, self-reflection frameworks, and human-in-the-loop validation, have shown considerable promise in improving the reliability of AI-generated medical information and reducing hallucinations in healthcare applications [94,95]. Despite these technological advances, healthcare remains a life-critical domain where incorrect recommendations may result in substantial patient harm. Therefore, GenAI should be regarded as a **decision-support tool** that complements, rather than replaces, clinical expertise, and AI-generated recommendations should continue to encourage consultation with qualified healthcare professionals before diagnostic or therapeutic decisions are made [92,93].

### Strengths and limitations of the study

The study adds to the body of knowledge about how individuals use automated assistance through technological advancements by assessing the many ways that people engage in self-medical care. Previous studies only focused on one aspect of self-care (e.g., self-medication) and did not examine all of the possible ways that new technologies, such as Generative AI, may assist people in making decisions related to their health. In addition, the study allows for the examination of the impact of educational and professional differences on the use of GenAI tools for health-related decision-making among students of higher educational institutions from various disciplines. Furthermore, the digital data collection tool using the Kobo Toolbox enhanced data quality, reduced errors when entering data, and allowed for the ability to monitor in real-time the status of the survey.

Despite this strength, it has limitations that should be considered while concluding the outcomes. First, the study included students from a single university, making it difficult to generalize the results to broader higher education students in Ethiopia. Second, recall bias may be introduced due to the evaluation of GenAI-assisted self-medical care based on the experiences of the participants over the 12 months before the survey. Finally, the study did not specifically differentiate self-medication practices related to antibiotics, which are typically prescription-only medications, and instead assessed medication use across all drug categories. This may have led to a higher estimate of self-medication for all prescriptions than actually occurs because some antimicrobial tools, such as some antiviral and antifungal drugs, may be available over the counter in some contexts.

## Conclusion and recommendations

The study demonstrated that GenAI-assisted medical self-care is highly prevalent among higher education students. The study also found that individual, technological, and behavioral factors influence the adoption of GenAI-assisted medical self-care, which includes an increase in year of study, awareness of GenAI technologies, positive attitude to GenAI technology, good digital literacy, positive health-seeking behavior, and health facility visits for recent illness.

Given a growing dependence on innovative technologies such as large language models and GenAI, universities, healthcare professionals, and policymakers should enhance digital health literacy and provide guidelines on the appropriate and safe use of GenAI in health care, while encouraging students to seek medical advice for conditions that require clinical evaluations. Dependence on GenAI for health decisions presents significant public health risks, including misdiagnosis, delayed treatment, and misinformation. To mitigate these risks, younger generations should develop strong digital health literacy to critically assess AI-generated outputs and evaluate these tools strictly as supplementary rather than diagnostic. Furthermore, GenAI Systems and AI technology companies should prioritize the safety, reliability, and transparency of health-related outputs through evidence-based practice, clear warnings about how to use the information, and embedded prompts that encourage users to seek qualified healthcare professionals for health issues that may arise as a result of serious medical events or if medications require medical supervision.

## Declarations

### Ethics approval and consent to participate

The study adhered to the principles of the Declaration of Helsinki, and ethical clearance was obtained from the College of Health Sciences, Arsi University Ethical Review Committee (Reference number: CoHS/R/239/2025/2026). Study participants were informed of the study’s purpose and provided informed consent. Participation in the study was voluntary, and participants who were unwilling to participate or wished to withdraw at any stage of the interview were allowed to do so without consequence. The confidentiality and privacy of the respondents were maintained.

## Supporting information

S1 Text Data collection tool/questionnaire. (PDF)

S1 Data Full dataset analyzed to generate the results reported in this study. (DTA)

## Abbreviations

AOR: Adjusted Odds Ratio
COR: Crude Odds Ratio
GenAI: Generative Artificial Intelligence
LLM: Large Language Model
LR: Loglikelihood Ratio
