# Supplementary material for: Generative artificial intelligence–assisted medical self-care and associated factors among undergraduate students at Arsi University, South-Eastern Ethiopia
Source: PLOS Digit Health. 2026 Sep 24;5(9):e0001748. doi: 10.1371/journal.pdig.0001748 (PMC13606965; doi:10.1371/journal.pdig.0001748)
Supplement: Supplementary file 1 [file pdig.0001748.s001.pdf]

Study Title: Utilization of Generative Artificial Intelligence Assisted Self-Care and Medication Guidance

The purpose of this study is to assess the use of generative artificial intelligence (GenAI) tools (ChatGPT, DeepSeek, Gemini, Copilot, and Perplexity) for self-care and medication guidance and to examine how this practice influences health care–seeking behavior among students at Public University in Ethiopia

---

If you agree to participate, you will be asked to complete a self-administered questionnaire. The questionnaire includes questions about your use of GenAI tools for health-related purposes, self-medication practices, attitudes toward AI-assisted self-care, and health care–seeking behaviors. Completing the questionnaire will take approximately 15–20 minutes.

Risks and Discomforts: The study involves minimal risk. Some questions may cause mild discomfort if they relate to personal health practices. You may skip any question you do not wish to answer.

Benefits: There is no direct benefit to you. However, the findings may contribute to improved understanding of digital health behaviors and support the development of policies and guidelines for the safe and effective use of GenAI in health care.

Confidentiality: All information collected will be kept strictly confidential. No personal identifiers will be recorded, and the data will be used only for research purposes. Voluntary Participation: Your participation is entirely voluntary. You may decline to participate or withdraw from the study at any time without any academic or personal consequences

---

Contact Information: If you have questions about the study, you may contact the principal investigator through: Email: adamjibrilbashir@gmail.com, Phone Number: +251922836268

Informed Consent Form: I have read and understood the information provided above. I have been informed about the purpose of the study, the procedures involved, potential risks and benefits, and my rights as a participant. I understand that my participation is voluntary and that I am free to withdraw from the study at any time without any negative consequences. By agreeing to participate, I confirm that: I am a student of public University in Ethiopia. I understand that my responses will be kept confidential and used only for research purposes

---

Do you agree to participate?

- (●) Yes
- ( ) No

SOCIODEMOGRAPHIC, SOCIOECONOMIC, AND ACADEMIC CHARACTERISTICS

Age in year

____

Sex

- ◯ Male
- ◯ Female

Marital status

- ◯ Single
- ◯ Married
- ◯ Other

Year of study

- ◯ First
- ◯ Second
- ◯ Third
- ◯ Fourth or above

Department/Field of study

____

Self-reported cumulative GPA

- ◯ <2.50
- ◯ 2.50–2.99
- ◯ 3.00–3.49
- ◯ ≥3.50

Average monthly financial support (ETB)

- ◯ <1000
- ◯ 1000–2999
- ◯ 3000–4999
- ◯ ≥5000

Primary source of financial support

- ◯ Family
- ◯ Scholarship
- ◯ Part-time
- ◯ Other

Place of upbringing

- ◯ Urban
- ◯ Rural

Current living arrangement

- ◯ University dormitory
- ◯ Off-campus rental
- ◯ With family

HEALTH STATUS, ILLNESS EXPERIENCE, AND MEDICATION HISTORY

Self-rated overall health status

- ◯ Poor
- ◯ Fair
- ◯ Good
- ◯ Very good

Do you have any chronic illness?

- ◯ Yes
- ◯ No

Number of illness episodes (past 12 months)

- ◯ None
- ◯ 1–2
- ◯ ≥3

Severity of most recent illness

- ◯ Mild
- ◯ Moderate
- ◯ Severe

Used prescribed medication in past 12 months?

- ◯ Yes
- ◯ No

Experienced adverse drug reaction?

- ◯ Yes
- ◯ No

LIFESTYLE AND PSYCHOSOCIAL FACTORS

Khat chewing habit

- ○ Never
- ○ Occasionally
- ○ Regularly

Alcohol consumption

- ○ Never
- ○ Occasionally
- ○ Regularly

Tobacco use

- ○ Never
- ○ Former user
- ○ Current user

Average nightly sleep duration

- ○ <5 hours
- ○ 5–7 hours
- ○ >7 hours

Perceived academic stress

- ○ Low
- ○ Moderate
- ○ High

How often do you tolerate symptoms before seeking help?

- ○ Never
- ○ Sometimes
- ○ Often

DIGITAL ACCESS AND HEALTH INFORMATION–SEEKING BEHAVIOR

Digital devices owned

- ☐ Smartphone
- ☐ Laptop
- ☐ Tablet

Reliability of internet access

- Poor
- Moderate
- Good

Average daily internet use

- <2 hours
- 2–4 hours
- >4 hours

Have you searched for health info online?

- Yes
- No

Frequency of searching health information

- Never
- Sometimes
- Often

Online health information sources

- Search engines/websites
- Social media platforms
- Online forums/communities
- Digital health applications
- GenAI tools

Primary reason for searching health information

- Understanding symptoms
- Deciding whether to seek care
- Medication information
- Prevention/lifestyle
- Academic curiosity

DIGITAL HEALTH LITERACY

I know where to find health information online

- Strongly disagree
- Disagree
- Neutral
- Agree
- Strongly agree

I know how to use the internet to answer health questions

- Strongly disagree
- Disagree
- Neutral
- Agree
- Strongly agree

I can judge whether online health information is reliable

- Strongly disagree
- Disagree
- Neutral
- Agree
- Strongly agree

I can understand online health information

- Strongly disagree
- Disagree
- Neutral
- Agree
- Strongly agree

I verify online information using more than one source

- Strongly disagree
- Disagree
- Neutral
- Agree
- Strongly agree

I know when online info should not replace professional care

- ◯ Strongly disagree
- ◯ Disagree
- ◯ Neutral
- ◯ Agree
- ◯ Strongly agree

GENAI AWARENESS

Have you heard of GenAI tools?

- ◯ Yes
- ◯ No

Which are GenAI tools?

- ☐ ChatGPT
- ☐ Gemini
- ☐ Copilot
- ☐ Google Search
- ☐ I do not know

GenAI tools primarily work by

- ◯ Predicting responses from large datasets
- ◯ Searching the internet only
- ◯ Copying from websites
- ◯ Do not know

GenAI health info may be incorrect

- ◯ True
- ◯ False
- ◯ Not sure

GENAI-BASED SELF-CARE AND SELF-MEDICATION PRACTICES

Used GenAI (ChatGPT, Deepsek, Gemini, Copilot and other) for self-care in past 12 months?

- ◉ Yes
- ◯ No

Type of guidance obtained

- [ ] Self-care advice
- [ ] Over-the-counter medicines
- [ ] Dosage/side effects
- [ ] Home remedies
- [ ] Lifestyle advice

Self-care actions practiced

- [ ] Symptom monitoring
- [ ] Rest and physical care
- [ ] Emotional coping
- [ ] Traditional remedies
- [ ] OTC medicines
- [ ] Lifestyle adjustments
- [ ] Seeking information

How often did you self-care before seeking care?

- ○ Rarely
- ○ Sometimes
- ○ Often
- ○ Always

HALTH CARE SERVICE ACCESS AND EXPERIENCE

Visited health facility during recent illness?

- ○ Yes
- ○ No

Time taken to seek care

- ○ Same day
- ○ 1–3 days
- ○ ≥4 days

Usual first action when unwell

- ◯ Wait/observe
- ◯ Self-manage
- ◯ Consult peers/family
- ◯ Visit facility

Overall facility use (past 12 months)

- ◯ Regular
- ◯ Occasional
- ◯ Rare

Distance to nearest health facility

- ◯ <30 min
- ◯ 30–60 min
- ◯ >60 min

Average waiting time

- ◯ Short
- ◯ Moderate
- ◯ Long

Affordability of care

- ◯ Affordable
- ◯ Moderately expensive
- ◯ Very expensive

Availability of essential medicines

- ◯ Adequate
- ◯ Sometimes inadequate
- ◯ Frequently unavailable

Trust in health professionals

- ◯ Low
- ◯ Moderate
- ◯ High

Overall satisfaction

- Low
- Moderate
- High

ATTITUDES TOWARD GENAI-ASSISTED SELF-CARE AND MEDICATION GUIDANCE

GenAI tools are useful for minor illness self-care

- Strongly disagree
- Disagree
- Neutral
- Agree
- Strongly agree

GenAI improves confidence in symptom management

- Strongly disagree
- Disagree
- Neutral
- Agree
- Strongly agree

GenAI influences my health decisions

- Strongly disagree
- Disagree
- Neutral
- Agree
- Strongly agree

I worry GenAI may provide incorrect information

- Strongly disagree
- Disagree
- Neutral
- Agree
- Strongly agree

GenAI use may lead to unsafe self-care

- Strongly disagree
- Disagree
- Neutral
- Agree
- Strongly agree

GenAI should not replace health professionals

- Strongly disagree
- Disagree
- Neutral
- Agree
- Strongly agree

GenAI should be regulated before medical use

- Strongly disagree
- Disagree
- Neutral
- Agree
- Strongly agree
