# Supplementary material for: Generative artificial intelligence–assisted medical self-care and associated factors among undergraduate students at Arsi University, South-Eastern Ethiopia
Source: PLOS Digit Health. 2026 Sep 24;5(9):e0001748. doi: 10.1371/journal.pdig.0001748 (PMC13606965; doi:10.1371/journal.pdig.0001748)
Supplement: Supplementary file 2 [file pdig.0001748.s004.pdf]

Date: May 14, 2026

Dear editorial board member (s) of PLOS Digital Health Journal

The authors have been recalled to revise the manuscript entitled **“Generative Artificial Intelligence–Assisted Medical Self-Care and Associated Factors among Undergraduate Students at Arsi University, Central Ethiopia",** and identified by Submission ID: PDIG-D-26-00433 under PLOS Digital Health Journal, which was submitted for publication. So, we received the editor’s and reviewers’ major revision comments for the improvement of the manuscript before it can be proceeded to publication.

Thank you for receiving comments, suggestions, directions, and questions from the editor and reviewer. As we said usually, we are very happy in receiving constructive and valuable comments for making a better manuscript. Accordingly, we have considered all the comments, questions, directions, and suggestions and provided a point-by-point response letter.

Finally, we have submitted all the required documents in their revised form. We hope that we have addressed all the suggestions, directions, and raised questions and if you believe that point(s) is not addressed, please let us know.

Thank you very much to editor (s), and reviewer

On the behalf of the authors

Yours sincerely,

Correspondence author

Point-by-point response letter

Editor comments

Journal Requirements:

1. Please provide an Author Summary. This should appear in your manuscript between the Abstract (if applicable) and the Introduction, and should be 150–200 words long. The aim should be to make your findings accessible to a wide audience that includes both scientists and non-scientists. Sample summaries can be found on our website under Submission Guidelines: [LINK]. https://journals.plos.org/digitalhealth/s/submission-guidelines#loc-parts-of-a-submission

Authors Response: Thank you for your comment we have added author summary between the Abstract (if applicable) and the Introduction (**See Page iii, Line number 68 - 83**).

2. Please provide separate figure files in .tif or .eps format. For more information about figure files please see our guidelines: LINK. https://journals.plos.org/digitalhealth/s/figures. https://journals.plos.org/digitalhealth/s/figures#loc-file-requirements.

Authors Response: We have improved the and uploaded separate figure (**See figure 1**).

3. In the online submission form, you indicated that "All relevant data are in the manuscript. However, the minimal data underlying all the findings in the manuscript will be available upon request to Jibril Bashir Adem.". All PLOS journals now require all data underlying the findings described in their manuscript to be freely available to other researchers, either
   1. In a public repository,
   2. Within the manuscript itself, or
   3. Uploaded as supplementary information.

This policy applies to all data except where public deposition would breach compliance with the protocol approved by your research ethics board. If your data cannot be made publicly available for ethical or legal reasons (e.g., public availability would compromise patient privacy), please explain your reasons by return email and your exemption request will be escalated to the editor for approval. Your exemption request will be handled independently and will not hold up the

peer review process, but will need to be resolved should your manuscript be accepted for publication. One of the Editorial team will then be in touch if there are any issues.

Author Response: Thank you for the comment; we have submitted the full data used in this study as supplementary file in the submission portal in Stata file extension (**See supplementary file 2**).

Reviewer 1 comments

This paper describes a survey of Ethiopian students, aimed at understanding patterns in the use of generative AI for health care. The study is well-motivated and situated in relevant work, the survey itself seems very straightforward, and the results are generally informative. The discussion is particularly clearly presented, with appropriate comparisons to prior work. There are some concerns with the exposition of the methods and the presentations of some of the results, but the paper is otherwise informative and worthwhile.

1. The two biggest concerns in this paper involve the description of the methods and some of the results. Regarding survey methods, we are told in section 2.7 that a "self-administered, structured questionnaire" was used, but no details are given. This section should be expanded to include details of the questionnaire. Ideally, the questionnaire should be provided as supplemental data.

Author Response: Thank you for this interesting comment; we have included the link to the data collection tool and uploaded the questionnaire as supplementary file 1 in the submission portal and cited it in the manuscript.

2. Regarding the analysis, additional detail is needed on both the binary and multivariable logistic regressions. For the binary regression, why such as large p-value (p <= 0.25)? For the multivariable analysis, which factors were included?

Author Response: We appreciate your feedback regarding the analysis section. We have made revisions to the analysis portion of our paper to better explain the bivariable and multivariable logistic regression analyses. The variables with a p-value ≤ 0.25 from the bivariable analysis were selected as potential candidates for inclusion in the multivariable model, which will prevent the exclusion of variables that may be important to the analysis and/or serve as confounders prior to any adjustment being made, as is recommended within the logistic regression modeling literature (Hosmer et al., 2013). We have also clearly specified all of the variables that were included in the multivariable logistic regression analysis and revised the final model description to enhance clarity and consistency (**See line number 249 - 258**).

3. In terms of the results, section 3.3 and Figure 1 are somewhat incongruous. Results are described as if Figure 1 includes the actions taken after consulting Gen-AI, but those results are not included in Figure 1. Furthermore, the pie chart is not a very useful way of providing the results.

Author Response: Thank you for this important comment, we have revised the figure and result interpretation for these specific sections and provided the updated figure in the revised document (**See line number: 244 – 247 and Figure 1**)

4. Section 3.4 discusses the results of the multivariable analysis, mentioning differences between the final model and the null model, without any definition of what those models were. This should be clarified. Similarly, Table 3 claims to have information on both bivariate and multivariate analyses, but it's not clear which is which. Details of the multivariable analysis need to be provided, and added to the interpretation.

Author Response: Thank you for the comment. We have revised this section by including the definition the final model and the null model in bracket for both. Also, we have separated the bivariate result as a Crude Odd Ratio (COR) with 95% Confidence Interval and Adjusted Odd Ratio (AOR) with 95% Confidence Interval for multivariate analyses result table (**See line number: 259 – 270 and Table 3**).

Minor concerns:

5. Section 2.1 - the study should be clearly described as being a survey.

Author Response: Thank you for this important comment; we have revised the study design section to clearly indicated the survey nature of our study (**See line number: 153 – 155)**.

6. A reference to the Kobo toolbox should be provided.

Author Response: Thank you for this important comment; we provided the link to Kobo toolbox in the manuscript (**See line number: 203 - 204)**

7. The abstract should be revised to clarify that the study is specific to students in Ethiopia.

Author Response: Thank you for the comment we have indicted clearly as the study was conducted the among specific students at Arsi University in Ethiopia in the Abstract (**See line number: 35 - 39).**

Reviewer 2 comments

1. You have comprehensively studied various aspects. There are a few shortcomings. Through this study, where are your inferences?

Author Response: We appreciate that you have provided us with such a helpful comment. We have made significant improvements to the interpretation and reasoning behind our findings through the discussion and conclusion sections of our paper. In addition, we have added further detail to describe how the prevalence of GenAI-assisted medical self-care affects individuals' behavior regarding their health, the influence of digital literacy in developing appropriate health behaviors, and how factors associated with the healthcare system could impact the uptake of self-care using AI. Finally, we have highlighted some of the important implications for public health associated with the increased use of GenAI components in making health decisions by university students, including the necessity for responsible use of AI, the importance of developing digital health literacy, and the need for proper consultation with health professionals when using AI-supported health information (**See Discussion and conclusion section**).

2. What are the technical and business advantages and disadvantages of generative AI tools?

Author Response: We are grateful for the valuable suggestion and have revised the discussion section to present the advantages and disadvantages of utilizing generative AI tools in a technical and business context in healthcare. In particular, we discuss the advantages associated with using GenAI tools, including providing rapid access to health information, continuous access to information, scalability, low cost of support, and personalized interaction; all of which could offer a broader base for obtaining health-related assistance for students and users of digital technology. We also provided detailed limitations of these tools, including the potential creation of inaccurate or misleading medical information, the need for the clinical validation of generative AI-based information, privacy and security of data, algorithmic bias, over-reliance on AI-generated recommendations, and lack of accountability for health-related decisions made using generative AI. Subsequently, we reiterated that commercial AI platforms may typically enhance user engagement and scalability, and do not place emphasis on the clinical safety of their users, which further reinforces the importance of increased regulatory oversight and transparency as well as the integration of safeguards that promote the consultation of professional healthcare providers.

3. As healthcare is a life-critical domain, is it prudent to use GenAI tools? What are the prominent generative AI tools (large language models) used by the people?

Author Response: We thank you for your valued comment. We have added to our discussion regarding both the possible benefits and possible risks associated with the use of generative models in healthcare. Healthcare is, after all, a domain where human lives can be affected. We now note that GenAI has the potential to provide more timely access to health information, assist in interpreting symptoms, provide enhanced health education, and improve access to healthcare resources in underserved areas. However, we also pointed out that the use of GenAI tools must be done with care because they may produce data that is inaccurate or misleading (also referred to as "hallucinated"), do not contain level of clinical reasoning that a healthcare professional

possesses, and may create ethical concerns with respect to privacy, bias, accountability, and the safety of patients (**See the discussion section**).
