# Supplementary material for: Generative artificial intelligence–assisted medical self-care and associated factors among undergraduate students at Arsi University, South-Eastern Ethiopia
Source: PLOS Digit Health. 2026 Sep 24;5(9):e0001748. doi: 10.1371/journal.pdig.0001748 (PMC13606965; doi:10.1371/journal.pdig.0001748)
Supplement: Supplementary file 3 [file pdig.0001748.s005.pdf]

Date: July 1, 2026

Dear editorial board members of PLOS Digital Health Journal

The authors have been recalled to revise the manuscript entitled **“Generative Artificial Intelligence–Assisted Medical Self-Care and Associated Factors among Undergraduate Students at Arsi University, Central Ethiopia",** and identified by Submission ID: PDIG-D-26-00433 under PLOS Digital Health Journal, which was submitted for publication. So, we received the reviewers' minor revision comments to improve the manuscript before it can proceed to publication.

Thank you for receiving comments, suggestions, directions, and questions from the editor and reviewer. As usual, we are very happy to receive constructive and valuable comments for making a better manuscript. Accordingly, we have considered all the comments, questions, directions, and suggestions and provided a point-by-point response letter.

Finally, we have submitted all the required documents in their revised form. We hope that we have addressed all the suggestions, directions, and raised questions, and if you believe that point(s) is not addressed, please let us know.

Thank you very much to the editor (s) and reviewer

On behalf of the authors

Yours sincerely,

Correspondence author

Point-by-point response letter

Reviewer 1 comments

Thank you for your revisions: this version is a notable improvement. There are two areas in need of improvement.

1. Section 3.4, the first paragraph lists the factors that were significantly associated with the outcome at the p-value of 0.25. The second paragraph (starting on line 294) lists variables that we included in the multivariate logistic regression. Shouldn't that be the same set of variables as those that we associated at p=0.25? This point should be clarified.

Author Response: Thank you for your constructive comment. We revised Section 3.4 by indicating that a binary logistic regression model was fitted to see the association between GenAI-assisted medical self-care and multiple independent variables using bivariate and multivariable analysis. In bivariate analysis, twenty variables were fitted, and twelve variables were found to be candidate variables and were included in the multivariable regression for having $p<=0.25$; finally, seven variables were found to be significantly associated with the outcome variable at $p<0.05$ (**Page number 7 - 8; line number: 251 -267**).

2. The discussion of the students' seniority is potentially misleading. Admittedly, the large odds ratio for second-year students is intriguing. However, this differential should be incorporated in the context of comparable odds ratios for years 3 and 4, which are not nearly as high. Focusing only on the differential for 2nd-year students (lines 321-323 and 420-421) overstates the effect. These passages should be revised accordingly.

Author Response: We thank you for your valued comment. We have revised the paragraph to explicitly indicate the significant student year group (2nd year) in the comparison and interpretation of the result within the related literature context (**Page 11 -12; line 338 - 352**).
