# Supplementary material for: Generative artificial intelligence–assisted medical self-care and associated factors among undergraduate students at Arsi University, South-Eastern Ethiopia
Source: PLOS Digit Health. 2026 Sep 24;5(9):e0001748. doi: 10.1371/journal.pdig.0001748 (PMC13606965; doi:10.1371/journal.pdig.0001748)
Supplement: Supplementary file 4 [file pdig.0001748.s006.pdf]

Date: August 3, 2026

Dear editorial board members of PLOS Digital Health Journal

The authors have been asked to revise the manuscript entitled **"Generative Artificial Intelligence–Assisted Medical Self-Care and Associated Factors among Undergraduate Students at Arsi University, Southeastern Ethiopia",** identified by Submission ID: PDIG-D-26-00433, under PLOS Digital Health Journal, which was submitted for publication. So, we received the reviewers' minor revision comments to improve the manuscript before it can proceed to publication.

Thank you for receiving comments, suggestions, directions, and questions from the editor and reviewer. As usual, we are very happy to receive constructive and valuable comments to help us improve the manuscript. Accordingly, we have considered all the comments, questions, directions, and suggestions and provided a point-by-point response letter.

Finally, we have submitted all the required documents in their revised form. We hope we have addressed all the suggestions, directions, and questions raised, and if you believe any point(s) are not addressed, please let us know.

Thank you very much to the editor (s) and reviewer

On behalf of the authors

Yours sincerely,

Correspondence author

Point-by-point response letter

Editor's comment

1. While the manuscript has been improved, the remaining concerns raised by the reviewer must be fully addressed. Additionally, please provide an analysis of any bias or stratified features within the collected data (e.g., distribution across sex, year of study, place of upbringing, or other demographic characteristics), and double-check the references (there are many typos).

Author Response: Thank you for this important comment. We have conducted an additional analysis to assess potential stratification patterns and sources of imbalance within the collected data. Specifically, the distribution of GenAI-assisted medical self-care utilization was examined across key sociodemographic, academic, and socioeconomic characteristics, including sex, age, marital status, year of study, field of study, GPA, income level, place of upbringing, source of income, and current living arrangement. Pearson's chi-square tests were performed to evaluate differences in GenAI-assisted medical self-care utilization across these subgroups. The additional analysis showed that GenAI-assisted medical self-care utilization was generally consistent across most demographic characteristics; however, significant variation was observed across year of study, with the highest utilization among second-year students. This stratified analysis has been added to the revised manuscript (Table 2) to provide greater transparency regarding sample characteristics and potential sources of bias.

Furthermore, all variables identified as theoretically relevant or potentially confounding factors were considered in the multivariable logistic regression analysis to minimize the influence of observed differences between participant groups. We have also carefully reviewed the reference list and corrected typographical errors, formatting inconsistencies, and citation-related issues throughout the manuscript. The new stratified analysis has been incorporated in the Results section under "Distribution of GenAI-assisted medical self-care utilization across participant characteristics" (**Page 7 - 8, Lines 253 - 265**), and the corresponding table has been added as Table 2.

First Reviewer's comment

Thanks for your responses to my comments.

1. I remain concerned that the response to my concern about the 2nd vs. 3rd and 4th year students misses the point of my comments and obscures a potentially interesting issue. Your response notes the increased likelihood of use among 2nd-year students. Still, it fails to discuss the apparent reversal of the trend, with much lower COR and AOR results among 3rd- and 4th-year students, including confidence intervals below the 1.0 baseline. These numbers suggest that there might be some interesting trends going on - perhaps there are some students who become less likely to use the AI tools over time. Although these changes might not be significant, they deserve mention because the situation is clearly more complex than simply a case of students past their first year having more exposure to health-related information in their courses.

Author Responses: Thank you for this insightful observation. We agree that our previous interpretation oversimplified the relationship between year of study and GenAI-assisted medical self-care. We have revised the discussion to acknowledge the apparent non-linear pattern observed across academic years. Specifically, while second-year students demonstrated significantly higher odds of GenAI-assisted medical self-care than first-year students, the crude and adjusted estimates for third- and fourth-year students were lower, suggesting that the relationship is more complex than a simple increase with academic progression. Although these differences were not statistically significant, we now discuss this pattern as a possible indication that reliance on GenAI tools may decline among senior students, potentially reflecting greater academic maturity, increased critical appraisal skills, greater reliance on formal clinical knowledge and healthcare professionals, or changing academic and clinical experiences. We have emphasized that these explanations are speculative and that longitudinal studies are needed to determine whether GenAI use changes over the course of university education **(Page number: 13, line number: 360 - 389).**

Second Reviewer's comments

1. Appreciate the authors for addressing all the concerns and clarifications raised by the reviewer. There are definitely some limitations on generative AI models. However, the hallucination problem is steadily being solved. Other viable technologies and tools can surmount the limitations of generative AI tools. The recent LLMs are more powerful and accurate in their decisions and planning. However, giving 100 percent control to AI systems is risky.

Author Response: We appreciate your insightful comment. We concur that generative AI technologies are advancing rapidly and that recent large language models have shown significant enhancements in reasoning accuracy, planning capabilities, and the reduction of hallucinations. In our revised discussion, we aim to present a more balanced viewpoint by recognizing these technological advancements and highlighting emerging strategies such as retrieval-augmented generation, integration with evidence-based knowledge sources, and the incorporation of human oversight, all of which contribute to the reliability of AI-generated health information. Nevertheless, we stress that, despite these improvements, healthcare remains a critical domain, and generative AI should function as a decision-support tool that complements, rather than substitutes, professional clinical judgment. The updated discussion is now supported by recent evidence from the literature (**Page 15 - 16; lines 444 - 457**).
