# Supplementary material for: Generative artificial intelligence–assisted medical self-care and associated factors among undergraduate students at Arsi University, South-Eastern Ethiopia
Source: PLOS Digit Health. 2026 Sep 24;5(9):e0001748. doi: 10.1371/journal.pdig.0001748 (PMC13606965; doi:10.1371/journal.pdig.0001748)
Supplement: Peer review history

# Peer review: Decision Letter 0

**DOI:** 10.1371/journal.pdig.0001748.r001

**Authors:** Dhiya Al-Jumeily OBE, Kezhi Li

14 May 2026

Response to Reviewers Revised Manuscript with Track Changes Manuscript **Journal Requirements:**

1. Please provide an Author Summary. This should appear in your manuscript between the Abstract (if applicable) and the Introduction, and should be 150–200 words long. The aim should be to make your findings accessible to a wide audience that includes both scientists and non-scientists. Sample summaries can be found on our website under Submission Guidelines: [LINK]

https://journals.plos.org/digitalhealth/s/submission-guidelines#loc-parts-of-a-submission

2. Please provide separate figure files in .tif or .eps format.

For more information about figure files please see our guidelines:  LINK

https://journals.plos.org/digitalhealth/s/figures

https://journals.plos.org/digitalhealth/s/figures#loc-file-requirements

3. In the online submission form, you indicated that "All relevant data are in the manuscript. However, the minimal data underlying all the findings in the manuscript will be available upon request to Jibril Bashir Adem.".

All PLOS journals now require all data underlying the findings described in their manuscript to be freely available to other researchers, either

1. In a public repository,

2. Within the manuscript itself, or

3. Uploaded as supplementary information.

This policy applies to all data except where public deposition would breach compliance with the protocol approved by your research ethics board. If your data cannot be made publicly available for ethical or legal reasons (e.g., public availability would compromise patient privacy), please explain your reasons by return email and your exemption request will be escalated to the editor for approval. Your exemption request will be handled independently and will not hold up the peer review process, but will need to be resolved should your manuscript be accepted for publication. One of the Editorial team will then be in touch if there are any issues.

**Additional Editor Comments (if provided):** **Reviewers' Comments:**

**Comments to the Author**

1. Does this manuscript meet PLOS Digital Health’s publication criteria?

Reviewer #1: Yes

Reviewer #2: Yes

**********

2. Has the statistical analysis been performed appropriately and rigorously?

Reviewer #1: I don't know

Reviewer #2: Yes

**********

3. Have the authors made all data underlying the findings in their manuscript fully available (please refer to the Data Availability Statement at the start of the manuscript PDF file)?

The PLOS Data policy

Reviewer #1: No

Reviewer #2: Yes

**********

4. Is the manuscript presented in an intelligible fashion and written in standard English?

Reviewer #1: Yes

Reviewer #2: Yes

**********

Reviewer #1: This paper describes a survey of Ethiopian students, aimed at

understanding patterns in the use of generative AI for health

care. The study is well-motivated and situated in relevant work, the

survey itself seems very straightforward, and the results are

generally informative. The discussion is particularly clearly

presented, with appropriate comparisons to prior work. There are some

concerns with the exposition of the methods and the presentations of

some of the results, but the paper is otherwise informative and

worthwhile.

The two biggest concerns in this paper involve the description of the

methods and some of the results. Regarding survey methods, we are told

in section 2.7 that a "self-administered, structured questionnaire" was

used, but no details are given. This section should be expanded to

include details of the questionnaire. Ideally, the questionnaire

should be provided as supplemental data.

Regarding the analysis, additional detail is needed on both the binary

and multivariable logistic regressions. For the binary regression, why

such as large p-value (p <= 0.25)? For the multivariable analysis,

which factors were included?

In terms of the results, section 3.3 and Figure 1 are somewhat

incongruous. Results are described as if Figure 1 includes the actions

taken after consulting Gen-AI, but those results are not included in

Figure 1. Furthermore, the pie chart is not a very useful way of

providing the results.

Section 3.4 discusses the results of the multivariable analysis,

mentioning differences between the final model and the null model,

without any definition of what those models were. This should be

clarified. Similarly, Table 3 claims to have information on both

bivariate and multivariate analyses, but it's not clear which is

which. Details of the multivariable analysis need to be provided, and

added to the interpretation.

Minor concerns:

1. Section 2.1 - the study should be clearly described as being a

survey.

2. A reference to the Kobo toolbox should be provided.

3. The abstract should be revised to clarify that the study is

specific to students in Ethiopia.

Reviewer #2: You have comprehensively studied various aspects. There are a few shortcomings. Through this study, whare are your inferences? What are the technical and business advantages and disadvantages of generative AI tools. As healthcare is a life-critical domain, is it prudent to use GenAI tools? What are the prominent generative AI tools (large language models) used by the people

**********

what does this mean?). If published, this will include your full peer review and any attached files.

**Do you want your identity to be public for this peer review?**  If you choose “no”, your identity will remain anonymous but your review may still be made public.

For information about this choice, including consent withdrawal, please see our Privacy Policy

Reviewer #1: **Yes:** Harry Hochheiser

Reviewer #2: **Yes:** Pethuru Raj Chelliah

**********

**Figure resubmission:**

**Reproducibility:**

To enhance the reproducibility of your results, we recommend that authors of applicable studies deposit laboratory protocols in protocols.io, where a protocol can be assigned its own identifier (DOI) such that it can be cited independently in the future. Additionally, PLOS ONE offers an option to publish peer-reviewed clinical study protocols. Read more information on sharing protocols at https://plos.org/protocols?utm_medium=editorial-email&utm_source=authorletters&utm_campaign=protocols

# Author comment: Author response to Decision Letter 1

**DOI:** 10.1371/journal.pdig.0001748.r002

19 May 2026

Attachment Submitted filename: Response to Reviewers.pdf

# Peer review: Decision Letter 1

**DOI:** 10.1371/journal.pdig.0001748.r003

**Authors:** Dhiya Al-Jumeily OBE, Kezhi Li

30 Jun 2026

Response to ReviewersRevised Manuscript with Track ChangesManuscript**Journal Requirements:** **Additional Editor Comments (if provided):** **Reviewers' Comments:**

**Comments to the Author**

Reviewer #1: (No Response)

Reviewer #2: All comments have been addressed

**********

publication criteria?

Reviewer #1: Yes

Reviewer #2: Yes

**********

3. Has the statistical analysis been performed appropriately and rigorously?

Reviewer #1: Yes

Reviewer #2: Yes

**********

4. Have the authors made all data underlying the findings in their manuscript fully available (please refer to the Data Availability Statement at the start of the manuscript PDF file)?

The PLOS Data policy

Reviewer #1: Yes

Reviewer #2: Yes

**********

5. Is the manuscript presented in an intelligible fashion and written in standard English?

Reviewer #1: Yes

Reviewer #2: Yes

**********

**Reviewer #1** : Thank you for your revisions: this version is a notable improvement.

There are two areas in need of improvement.

1. Section 3.4 the first paragraph lists the factors that were significantly associated with the outcome at the p-value of 0.25. The second paragraph ( starting on line 294) lists variables that we included in the multivariate logistic regression. Shouldn't that be the same set of variables as those that we associated at p=0.25? This point should be clarified.

2. The discussion of the students' seniority is potentially misleading. Admittedly, the large odds-ratio for second-year students is intriguing. However, this differential should be incorporated in the context of comparable odds ratios for years 3 and 4, which are not nearly as high. Focusing only on the differential for 2nd-year students (lines 321-323 and 420-421) overstates the effect. These passages shouold be revised accordingly.

**Reviewer #2:** You have addressed all the concerns and clarifications raised by different reviewers. The revised version seems to be OK

**********

what does this mean?). If published, this will include your full peer review and any attached files.

**Do you want your identity to be public for this peer review?**  If you choose “no”, your identity will remain anonymous but your review may still be made public.

For information about this choice, including consent withdrawal, please see our Privacy Policy

Reviewer #1: **Yes:** Harry Hochheiser

Reviewer #2: **Yes:** Pethuru Raj Chelliah

**********

**Figure resubmission:**

**Reproducibility:**

To enhance the reproducibility of your results, we recommend that authors of applicable studies deposit laboratory protocols in protocols.io, where a protocol can be assigned its own identifier (DOI) such that it can be cited independently in the future. Additionally, PLOS ONE offers an option to publish peer-reviewed clinical study protocols. Read more information on sharing protocols at https://plos.org/protocols?utm_medium=editorial-email&utm_source=authorletters&utm_campaign=protocols

# Author comment: Author response to Decision Letter 2

**DOI:** 10.1371/journal.pdig.0001748.r004

1 Jul 2026

Attachment Submitted filename: Reponse to reviewers revision comments.pdf

# Peer review: Decision Letter 2

**DOI:** 10.1371/journal.pdig.0001748.r005

**Authors:** Dhiya Al-Jumeily OBE, Kezhi Li

2 Aug 2026

Response to ReviewersRevised Manuscript with Track ChangesManuscript**Journal Requirements:** **Additional Editor Comments (if provided):** **Reviewers' Comments:**

**Comments to the Author**

Reviewer #1: (No Response)

Reviewer #2: All comments have been addressed

**********

publication criteria?

Reviewer #1: Yes

Reviewer #2: Yes

**********

3. Has the statistical analysis been performed appropriately and rigorously?

Reviewer #1: Yes

Reviewer #2: Yes

**********

4. Have the authors made all data underlying the findings in their manuscript fully available (please refer to the Data Availability Statement at the start of the manuscript PDF file)?

The PLOS Data policy

Reviewer #1: Yes

Reviewer #2: Yes

**********

5. Is the manuscript presented in an intelligible fashion and written in standard English?

Reviewer #1: Yes

Reviewer #2: Yes

**********

**Reviewer #1:**  Thanks for your responses to my comments.

I remain concern that the response to my concern about the 2nd vs. 3rd and 4th year students misses the point of my comments and obscures a potentially interesting issue.

Your response notes the increased likelihood of use for 2nd year students, but it fails to discuss the seeming reversal of the trend, with much lower COR and AOR results for 3rd and 4th year students, including confidence intervals below the 1.0 baseline. These numbers suggest that there might be some interesting trends going on - perhaps there are some students who become less likely to use the AI tools over time. Although these changes might not be significant, they deserve mention, because the situation is clearly more complex than simply a case of students past their first year having more exposure to health-related information in their courses.

**Reviewer #2:**  Appreciate the authors for addressing all the concerns and clarifications raissed by the reviewer. There are definitely some limitations on generative AI models. However, the hallucination problem is steadily solved. There are other viable technologies and tools tht can surmount the limiations of generative AI tools. The recent LLMs are more powerful and accuracy in their decisions and planning. However, giving 100 percent control to AI systems is risky

**********

what does this mean?). If published, this will include your full peer review and any attached files.

**Do you want your identity to be public for this peer review?**  If you choose “no”, your identity will remain anonymous but your review may still be made public.

For information about this choice, including consent withdrawal, please see our Privacy Policy

Reviewer #1: **Yes:**

Reviewer #2: **Yes:** Pethuru Raj Chelliah

**********

**Figure resubmission:**

**Reproducibility:**

To enhance the reproducibility of your results, we recommend that authors of applicable studies deposit laboratory protocols in protocols.io, where a protocol can be assigned its own identifier (DOI) such that it can be cited independently in the future. Additionally, PLOS ONE offers an option to publish peer-reviewed clinical study protocols. Read more information on sharing protocols at https://plos.org/protocols?utm_medium=editorial-email&utm_source=authorletters&utm_campaign=protocols

# Author comment: Author response to Decision Letter 3

**DOI:** 10.1371/journal.pdig.0001748.r006

3 Aug 2026

Attachment Submitted filename: Responses to the fourth draft comments.pdf

# Peer review: Decision Letter 3

**DOI:** 10.1371/journal.pdig.0001748.r007

**Authors:** Dhiya Al-Jumeily OBE, Kezhi Li

7 Sep 2026

Generative Artificial Intelligence–Assisted Medical Self-Care and Associated Factors among Undergraduate Students at Arsi University, Southeastern Ethiopia

PDIG-D-26-00433R3

Dear Mr Adem,

We are pleased to inform you that your manuscript 'Generative Artificial Intelligence–Assisted Medical Self-Care and Associated Factors among Undergraduate Students at Arsi University, Southeastern Ethiopia' has been provisionally accepted for publication in PLOS Digital Health.

Before your manuscript can be formally accepted you will need to complete some formatting changes, which you will receive in a follow-up email from a member of our team.

Please note that your manuscript will not be scheduled for publication until you have made the required changes, so a swift response is appreciated.

IMPORTANT: The editorial review process is now complete. PLOS will only permit corrections to spelling, formatting or significant scientific errors from this point onwards. Requests for major changes, or any which affect the scientific understanding of your work, will cause delays to the publication date of your manuscript.

If your institution or institutions have a press office, please notify them about your upcoming paper to help maximize its impact. If they'll be preparing press materials, please inform our press team as soon as possible -- no later than 48 hours after receiving the formal acceptance. Your manuscript will remain under strict press embargo until 2 pm Eastern Time on the date of publication. For more information, please contact digitalhealth@plos.org.

Thank you again for supporting Open Access publishing; we are looking forward to publishing your work in PLOS Digital Health.

Best regards,

Kezhi Li, PhD

Section Editor

PLOS Digital Health

***********************************************************

**Additional Editor Comments (if provided):**

**Reviewer Comments (if any, and for reference):**

Reviewer's Responses to Questions

**Comments to the Author**

Reviewer #1: All comments have been addressed

Reviewer #2: All comments have been addressed

**********

publication criteria?

Reviewer #1: (No Response)

Reviewer #2: Yes

**********

3. Has the statistical analysis been performed appropriately and rigorously?

Reviewer #1: (No Response)

Reviewer #2: Yes

**********

4. Have the authors made all data underlying the findings in their manuscript fully available (please refer to the Data Availability Statement at the start of the manuscript PDF file)?

The PLOS Data policy

Reviewer #1: (No Response)

Reviewer #2: Yes

**********

5. Is the manuscript presented in an intelligible fashion and written in standard English?

Reviewer #1: (No Response)

Reviewer #2: Yes

**********

Reviewer #1: (No Response)

Reviewer #2: The authors have meticulously addressed all the concerns expressed by different reviewers. The paper is in good shape now

**********

what does this mean?). If published, this will include your full peer review and any attached files.

**Do you want your identity to be public for this peer review?**  If you choose “no”, your identity will remain anonymous but your review may still be made public.

For information about this choice, including consent withdrawal, please see our Privacy Policy

Reviewer #1: None

Reviewer #2: **Yes:** Pethuru Raj Chelliah

**********
